# Vitamin D intake and risk of CVD and all-cause mortality: evidence from the Caerphilly Prospective Cohort Study

**DOI:** 10.1017/S1368980017001732

**Published:** 2017-08-14

**Authors:** Jing Guo, John R Cockcroft, Peter C Elwood, Janet E Pickering, Julie A Lovegrove, David I Givens

**Affiliations:** 1 Institute for Food, Nutrition and Health, University of Reading, Reading RG6 6AR, UK; 2 Wales Heart Research Institute, Cardiff University, Cardiff, UK; 3 Department of Primary Care and Public Health, Cardiff University, Cardiff, UK; 4 Hugh Sinclair Unit of Human Nutrition and Institute for Cardiovascular and Metabolic Research, Faculty of Life Sciences, University of Reading, Reading, UK

**Keywords:** Vitamin D, CVD, All-cause mortality, Caerphilly Prospective Cohort Study, TAG

## Abstract

**Objective:**

Prospective data on the associations between vitamin D intake and risk of CVD and all-cause mortality are limited and inconclusive. The aim of the present study was to investigate the associations between vitamin D intake and CVD risk and all-cause mortality in the Caerphilly Prospective Cohort Study.

**Design:**

The associations of vitamin D intake with CVD risk markers were examined cross-sectionally at baseline and longitudinally at 5-year, 10-year and >20-year follow-ups. In addition, the predictive value of vitamin D intake for CVD events and all-cause mortality after >20 years of follow-up was examined. Logistic regression and general linear regression were used for data analysis.

**Setting:**

Participants in the UK.

**Subjects:**

Men (*n* 452) who were free from CVD and type 2 diabetes at recruitment.

**Results:**

Higher vitamin D intake was associated with increased HDL cholesterol (*P*=0·003) and pulse pressure (*P*=0·04) and decreased total cholesterol:HDL cholesterol (*P*=0·008) cross-sectionally at baseline, but the associations were lost during follow-up. Furthermore, higher vitamin D intake was associated with decreased concentration of plasma TAG at baseline (*P*=0·01) and at the 5-year (*P*=0·01), but not the 10-year examination. After >20 years of follow-up, vitamin D was not associated with stroke (*n* 72), myocardial infarctions (*n* 142), heart failure (*n* 43) or all-cause mortality (*n* 281), but was positively associated with increased diastolic blood pressure (*P*=0·03).

**Conclusions:**

The study supports associations of higher vitamin D intake with lower fasting plasma TAG and higher diastolic blood pressure.

Recently, the UK Scientific Advisory Committee on Nutrition^(^ ^1^ ^)^ reported that 22–24 % of individuals aged 19–64 years and 17–24 % of those aged ≥65 years in the UK were vitamin D deficient (plasma 25-hydroxycholecalciferol <25 nmol/l). People generally obtain vitamin D from synthesis in the skin due to sunlight UV radiation and/or foods. However, a number of relatively recent lifestyle changes (increased working indoors, sunscreen use), personal characteristics (ageing, skin pigmentation) and geographic reasons (latitude) limit the ability to synthesise adequate vitamin D from sunlight^(^ ^2^ ^)^. As a result, vitamin D intake from foods has become more important than previously. This led the Scientific Advisory Committee on Nutrition to recommend a daily vitamin D intake of 10 µg for all adults within the UK^(^ ^1^ ^)^.

CVD is one of the main causes of morbidity and mortality in the world and there is mounting evidence indicating an association between suboptimal vitamin D status and increased risk of CVD^(^ ^3^ ^–^ ^6^ ^)^ and all-cause mortality^(^ ^7^ ^–^ ^9^ ^)^. However, few prospective cohort studies have analysed the relationship between vitamin D intake and CVD risk and all-cause mortality. In a 10-year cohort of 361 men and 394 women^(^ ^10^ ^)^, lower vitamin D intake was associated with an increased risk of stroke, but not myocardial infraction (MI). Furthermore, higher vitamin D intake was found to be associated with increased risk of CVD in a large US cohort of 74 272 women and 44 592 men^(^ ^11^ ^)^. In contrast, it was reported by two further prospective cohort studies of 34 486 postmenopausal Iowa women^(^ ^12^ ^)^ and 1529 Australians^(^ ^13^ ^)^ that vitamin D intake was not associated with CVD mortality.

The evidence on the association of vitamin D intake with CVD risk or all-cause mortality from prospective cohort studies is limited; we therefore investigated the associations between vitamin D intake and CVD events, CVD risk markers and all-cause mortality using the Caerphilly Prospective Cohort Study (CAPS) which has over 20 years of follow-up.

## Methods

### Study population

CAPS was initially set up in 1979–1983 to investigate the CVD risk factors based on 2512 men (45–59 years), representing 89 % of the subjects living in Caerphilly and the adjacent area^(^ ^14^ ^)^, who were followed up at 5-year intervals. At Phase 2 (1984–1988), 561 men were lost from Phase 1 (1979–1983) and an additional 447 men were recruited to follow up. In Phase 1, a representative 30 % sub-sample of subjects (665 men) was randomly selected at baseline to complete a 7 d weighed dietary intake record^(^ ^15^ ^)^. Food items were coded according to McCance and Widdowson^(^ ^16^ ^)^. These men were given weighing scales with instructions on how to complete the weighed dietary intake for seven consecutive days. From these records vitamin D intake was estimated based on food composition data given by McCance and Widdowson^(^ ^16^ ^)^; additionally, several manufacturers were contacted to obtain more information on new foodstuffs containing mixtures of ingredients^(^ ^15^ ^,^ ^17^ ^)^. To ensure consistency of the subject group throughout the study, the 134 men from the weighed intake subgroup who dropped out after Phase 1 were excluded from the current analysis. In addition, seventeen subjects who previously had a heart attack and sixty-two subjects with missing confounding factor data were excluded. Therefore, a total of 452 subjects were available for the current analysis.

### CVD events and all-cause mortality

Identification of stroke and vascular disease events has been described elsewhere^(^ ^18^ ^,^ ^19^ ^)^. Symptoms and illnesses suggestive a heart attack or stroke were confirmed according to the London School of Hygiene chest pain questionnaire and the Oxford Stroke Questionnaire. In addition, subjects were invited to clinics for an electrocardiogram measurement. Identification of fatal and non-fatal vascular disease events (ICD-10 (International Classification of Diseases, 10th revision) codes 121–5) including MI, heart failure and stroke (ICD-10 codes 163–4) were diagnosed by two independent expert clinicians and an epidemiologist using clinical evidence of computed tomography, pathological and radiological information. Furthermore, incidents of all-cause mortality were censored by the National Health Service Central Registry. Cause of death was defined according to ICD-9 (International Classification of Diseases, 9th revision).

### Cardiovascular risk markers

Fasting blood samples were taken at baseline, 5 years and 10 years to measure a wide range of CVD risk markers^(^ ^14^ ^)^. Plasma glucose and TAG were measured at baseline, 5-year and 10-year examinations; however, the ratio of total cholesterol (TC) to HDL cholesterol (HDL-C) and insulin were measured only at baseline and the 5-year examination.

After over 20 years of follow-up (until present), the haemodynamic variables of systolic blood pressure (SBP), diastolic blood pressure (DBP), aortic pulse wave velocity, augmentation index and mean arterial pressure were measured as described in detail elsewhere^(^ ^20^ ^,^ ^21^ ^)^. SBP and DBP were measured at each visit: baseline, 5 years, 10 years and 20 years^(^ ^14^ ^)^. Pulse pressure was calculated by subtracting DBP from SBP. The Friedewald formula^(^ ^22^ ^)^ was used to calculate LDL cholesterol.

### Statistical analyses

Data were analysed using the statistical software package Stata version 13.0 (2014). Our primary analysis investigated the longitudinal relationship between baseline vitamin D intake and CVD events (including stroke, MI and heart failure) and all-cause mortality after over 20 years of follow-up. Analyses of baseline vitamin D intake with CVD risk markers cross-sectionally at baseline, and longitudinally at 5-year or 10-year follow-up, were the secondary analyses. The subjects were divided into four groups according to vitamin D intake. Logistic regression and general linear regression statistical models were used to investigate the relationships of vitamin D intake with categorical and continuous variables of CVD risk markers, respectively. In addition, logistic regression analyses were used to estimate the OR of stroke, MI, heart failure and all-cause mortality. The first multivariate-adjusted model for all analyses included the confounding factors of age (years), BMI (kg/m^2^), social class (manual worker; non-manual worker), smoking (current smoker; never smoked; ex-smoker), leisure activity (with heavy work or exercise in leisure time; without heavy work or exercise in leisure time), alcohol (as ethanol, ml/week) and food energy intake (MJ/d). In addition, as vitamin D is functionally closely related with Ca, the second multivariate-adjusted model was further adjusted for Ca intake. For skewed variables, original data were transformed to natural logarithms for the regression model. The *β* coefficient was used to represent the change in the dependent variable for a 1 sd change in the exposure. Results were considered statistically significant at *P*<0·05.

## Results

The characteristics of the 452 subjects at baseline are shown in Table 1. The mean vitamin D intake was 21·0 (sd 19·3) µg/week. Subjects in the lowest quartiles of vitamin D intake were significantly more likely to be smokers (*P*=0·001) and had lower food energy intake (*P*<0·001). After controlling for total energy intake from foods, those with the highest weekly vitamin D intake tended to have higher intakes of fat (*P*=0·002), cereal fibre (*P*<0·001), vegetable fibre (*P*<0·001) and Ca (*P*<0·001). There were no associations between vitamin D intake and age, BMI, social class, leisure activity, alcohol consumption, protein intake and carbohydrate intake.

**Table 1:** Baseline characteristics (*n* 452) of participants by category of vitamin D intake, Caerphilly Prospective Cohort Study

	Vitamin D intake from foods (µg/week)								
	0·1–9·9		10·0–15·1		15·2–27·2		≥27·3		
Characteristic	Mean	sd	Mean	sd	Mean	sd	Mean	sd	*P* for trend
Subjects, (*n*)	114	–	112	–	114	–	112	–	
Age (years)	52	4·5	52	4·1	51	4·4	52	4·6	0·74
BMI (kg/m^2^)	26·3	3·1	25·9	3·1	26·3	3·6	26·1	3·1	0·78
Leisure activity (%)	43·9	–	52·7	–	50·0	–	57·1	–	0·08
Manual workers (%)	68·4	–	49·1	–	63·2	–	70·5	–	0·33
Current smokers (%)	64·9	–	58·0	–	50·9	–	44·6	–	0·001
Energy intake (MJ/d)	6·5	1·7	7·3	1·6	7·3	1·6	7·6	1·5	<0·001
Fat (% of food energy)	36·0	6·2	36·5	6·0	38·1	4·8	37·9	5·1	0·002
Protein (% of food energy)*	13·9	2·7	13·5	2·3	13·8	2·4	14·0	2·4	0·25
Carbohydrate (% of food energy)*	45·7	8·2	46·1	7·5	46·6	5·8	45·0	6·6	0·60
Alcohol intake (ml ethanol/week)†	37·0	62·7	32·2	38·7	21·7	27·7	29·0	35·0	0·11
Fibre, vegetable sources (g/d)†	8·2	0·3	8·4	0·2	8·5	0·3	8·6	0·3	<0·001
Fibre, cereal sources (g/d)†	7·3	1·4	7·9	1·1	8·1	1·2	9·1	1·2	<0·001
Ca intake (mg/week)†	5567	268	6343	200	6335	225	6613	224	<0·001

* Original data were transformed to natural logarithms for regression model.

† Data were adjusted for energy intake from foods.

Associations of vitamin D intake with different CVD risk markers were investigated at baseline as a cross-sectional analysis. There was a significant positive association between vitamin D intake and HDL-C (adjusted model 1: *P*=0·002; adjusted model 2: *P*=0·003; Table 2), with subjects consuming the highest vitamin D intake (≥27·3 µg/week) having 0·13 mmol/l higher mean HDL-C levels compared with subjects consuming the lowest vitamin D intake (0·1–9·9 µg/week). In addition, negative associations were observed between vitamin D intake and TC:HDL-C (adjusted model 1: *P*=0·005; adjusted model 2: *P*=0·008) and TAG concentration (adjusted model 1: *P*=0·003; adjusted model 2: *P*=0·01), with the subjects consuming ≥27·3 µg vitamin D/week having 0·7 mmol/l:mmol/l lower mean TC:HDL-C and 0·5 mmol/l lower mean plasma TAG compared with subjects consuming the lowest vitamin D intake (0·1–9·9 µg/week). Also, a modest positive trend was found between vitamin D intake and pulse pressure (adjusted model 1: *P*=0·03; adjusted model 2: *P*=0·04).

**Table 2:** Cross-sectional analysis between baseline vitamin D intake and markers of CVD risk, Caerphilly Prospective Cohort Study

	Vitamin D intake from foods (µg/week)				
Characteristic	0·1–9·9	10·0–15·1	15·2–27·2	≥27·3	*P* for trend
Total cholesterol					
Participants (*n*)	114	112	114	112	
Mean (mmol/l)	5·98	5·99	5·93	5·81	
sd	1·11	1·56	1·08	0·94	
Unadjusted coeff.	Ref.	0·001	0·053	0·174	0·25
Multivariable-adjusted *β* coeff.*	Ref.	0·012	0·025	0·109	0·49
Multivariable-adjusted *β* coeff.†	Ref.	0·029	0·007	0·083	0·59
HDL cholesterol					
Participants (*n*)	112	110	113	111	
Mean (mmol/l)	1·29	1·32	1·40	1·42	
sd	0·37	0·34	0·40	0·41	
Unadjusted coeff.	Ref.	0·025	0·104	0·122	0·006
Multivariable-adjusted *β* coeff.*	Ref.	0·023	0·129	0·134	0·002
Multivariable-adjusted *β* coeff.†	Ref.	0·020	0·126	0·131	0·003
Total cholesterol:HDL cholesterol‡					
Participants (*n*)	112	110	113	111	
Mean (mmol/l:mmol/l)	5·13	4·83	4·53	4·43	
sd	2·40	1·92	1·35	1·42	
Unadjusted coeff.	Ref.	0·045	0·094	0·120	0·004
Multivariable-adjusted *β* coeff.*	Ref.	0·039	0·102	0·112	0·005
Multivariable-adjusted *β* coeff.†	Ref.	0·035	0·098	0·107	0·008
LDL cholesterol					
Participants· (*n*)	112	108	103	109	
Mean (mmol/l)	4·30	4·28	4·21	4·09	
sd	1·12	1·51	1·08	0·98	
Unadjusted coeff.	Ref.	0·017	0·087	0·202	0·18
Multivariable-adjusted *β* coeff.*	Ref.	0·018	0·089	0·159	0·30
Multivariable-adjusted *β* coeff.†	Ref.	0·007	0·077	0·142	0·35
TAG‡					
Participants (*n*)	112	108	113	110	
Mean (mmol/l)	2·06	1·81	1·62	1·56	
sd	1·20	1·21	0·91	0·98	
Unadjusted coeff.	Ref.	0·130	0·206	0·262	<0·001
Multivariable-adjusted *β* coeff.*	Ref.	0·098	0·163	0·214	0·003
Multivariable-adjusted *β* coeff.†	Ref.	0·075	0·138	0·180	0·01
Systolic blood pressure					
Participants (*n*)	114	112	114	112	
Mean (mmHg)	140·5	139·8	141·6	140·6	
sd	20·9	18·1	20·1	17·0	
Unadjusted coeff.	Ref.	0·678	1·114	0·045	0·81
Multivariable-adjusted *β* coeff.*	Ref.	0·934	2·994	1·123	0·49
Multivariable-adjusted *β* coeff.†	Ref.	1·066	3·136	1·321	0·45
Diastolic blood pressure					
Participants (*n*)	114	112	114	112	
Mean, mmHg	89·4	90·1	88·7	87·4	
sd	12·0	11·7	12·4	11·9	
Unadjusted coeff.	Ref.	0·615	0·754	2·064	0·14
Multivariable-adjusted *β* coeff.*	Ref.	1·237	0·471	1·935	0·15
Multivariable-adjusted *β* coeff.†	Ref.	1·567	0·113	1·438	0·25
Pulse pressure					
Participants (*n*)	114	112	114	112	
Mean (mmHg)	51·1	49·8	53·0	53·2	
sd	14·5	13·6	13·2	13·8	
Unadjusted coeff.	Ref.	1·293	1·868	2·109	0·10
Multivariable-adjusted *β* coeff.*	Ref.	0·303	3·465	3·058	0·03
Multivariable-adjusted *β* coeff.†	Ref.	0·501	3·250	2·760	0·04

Coeff., coefficient; ref., reference category.

* Multivariable-adjusted model 1, adjusted for age, BMI, social class (manual and non-manual workers), alcohol intake (non-drinker, drinker has been divided into three equal groups), smokers (non-smoker, current smoker, previous smoker), leisure activity (yes and no) and food energy intake.

† Multivariable-adjusted model 2, additionally adjusted for Ca intake.

‡ Original data were transformed to natural logarithms for regression model.

In the longitudinal analyses of vitamin D intake and CVD risk markers at the 5-year examination, higher vitamin D intake was significantly negatively associated with plasma TAG concentration (adjusted model 1: *P*=0·003; adjusted model 2: *P*=0·01; Table 3). Subjects consuming the highest vitamin D intake (≥27·3 µg/week) had 0·48 mmol/l lower mean plasma TAG than subjects consuming the lowest vitamin D intake (0·1–9·9 µg/week). There were no significant associations between vitamin D intake and other CVD risk markers at the 5-year examination. In the longitudinal analyses at the 10-year examination, there were no associations between vitamin D intake and CVD risk markers (Table 4).

**Table 3:** Longitudinal analysis between baseline (Phase 1) vitamin D intake and markers of CVD risk after 5 years of follow-up, Caerphilly Prospective Cohort Study

	Vitamin D intake from foods (µg/week)				
Characteristic	0·1–9·9	10·0–15·1	15·2–27·2	≥27·3	*P* for trend
Total cholesterol					
Participants (*n*)	109	110	111	109	
Mean (mmol/l)	5·67	5·62	5·68	5·60	
sd	0·96	1·08	0·97	0·84	
Unadjusted coeff.	Ref.	−0·045	0·018	−0·068	0·73
Multivariable-adjusted *β* coeff.*	Ref.	−0·018	0·042	−0·025	0·98
Multivariable-adjusted *β* coeff.†	Ref.	0·027	0·086	0·038	0·69
HDL cholesterol					
Participants (*n*)	109	110	111	109	
Mean (mmol/l)	0·98	1·03	1·06	1·00	
sd	0·26	0·23	0·23	0·26	
Unadjusted coeff.	Ref.	0·046	0·078	0·018	0·42
Multivariable-adjusted *β* coeff.*	Ref.	0·034	0·08	0·010	0·46
Multivariable-adjusted *β* coeff.†	Ref.	0·034	0·08	0·011	0·47
Total cholesterol:HDL cholesterol‡					
Participants (*n*)	109	110	111	109	
Mean (mmol/l:mmol/l)	6·23	5·77	5·66	5·98	
sd	2·24	1·75	1·75	1·73	
Unadjusted coeff.	Ref.	−0·070	−0·084	−0·030	0·42
Multivariable-adjusted *β* coeff.*	Ref.	−0·051	−0·081	−0·015	0·56
Multivariable-adjusted *β* coeff.†	Ref.	−0·044	−0·073	−0·004	0·75
LDL cholesterol					
Participants (*n*)	109	110	111	109	
Mean (mmol/l)	4·23	4·17	4·28	4·24	
sd	0·90	0·99	0·96	0·81	
Unadjusted coeff.	Ref.	−0·062	0·047	0·010	0·72
Multivariable-adjusted *β* coeff.*	Ref.	−0·051	−0·081	−0·015	0·56
Multivariable-adjusted *β* coeff.†	Ref.	−0·044	−0·073	−0·004	0·75
TAG‡					
Participants (*n*)	109	110	111	109	
Mean (mmol/l)	2·27	2·13	1·74	1·79	
sd	1·66	1·41	0·77	1·05	
Unadjusted coeff.	Ref.	−0·078	−0·194	−0·197	0·001
Multivariable-adjusted *β* coeff.*	Ref.	−0·049	−0·172	−0·173	0·003
Multivariable-adjusted *β* coeff.†	Ref.	−0·032	−0·154	−0·148	0·01
Systolic blood pressure					
Participants (*n*)	113	109	113	111	
Mean (mmHg)	148·9	146·8	144·9	145·5	
sd	26·8	0·3	22·6	21·2	
Unadjusted coeff.	Ref.	−2·032	−3·973	−3·417	0·21
Multivariable-adjusted *β* coeff.*	Ref.	−0·233	−2·185	−2·083	0·40
Multivariable-adjusted *β* coeff.†	Ref.	−0·201	−2·15	−2·034	0·42
Diastolic blood pressure					
Participants (*n*)	113	109	113	111	
Mean, mmHg	86·2	84·3	84·1	83·0	
sd	12·3	10·7	10·9	11·9	
Unadjusted coeff.	Ref.	−1·873	−2·133	−3·194	0·04
Multivariable-adjusted *β* coeff.*	Ref.	−1·475	−1·865	−2·793	0·08
Multivariable-adjusted *β* coeff.†	Ref.	−1·340	−1·718	−2·587	0·11
Pulse pressure					
Participants (*n*)	113	109	113	111	
Mean (mmHg)	62·7	62·5	60·8	62·4	
sd	20·0	16·1	18·2	16·0	
Unadjusted coeff.	Ref.	−0·159	−1·84	−0·222	0·75
Multivariable-adjusted *β* coeff.*	Ref.	1·242	−0·320	0·711	0·94
Multivariable-adjusted *β* coeff.†	Ref.	1·139	−0·432	0·553	1·00

Coeff., coefficient; ref.; reference category.

* Multivariable-adjusted model 1, adjusted for age, BMI, social class (manual and non-manual workers), alcohol intake (non-drinker, drinker has been divided into three equal groups), smokers (non-smoker, current smoker, previous smoker), leisure activity (yes and no) and food energy intake.

† Multivariable-adjusted model 2, additionally adjusted for Ca intake.

‡ Original data were transformed to natural logarithms for regression model.

**Table 4:** Longitudinal analysis between baseline vitamin D intake and markers of CVD risk after 10 years of follow-up, Caerphilly Prospective Cohort Study

	Vitamin D intake from foods (µg/week)				
Characteristic	0·1–9·9	10·0–15·1	15·2–27·2	≥27·3	*P* for trend
TAG‡					
Participants (*n*)	85	98	99	99	
Mean (mmol/l)	2·13	2·08	1·80	1·78	
sd	1·42	1·52	0·90	0·90	
Unadjusted coeff.	Ref.	−0·048	−0·125	−0·132	0·06
Multivariable-adjusted *β* coeff.*	Ref.	−0·018	−0·102	−0·101	0·11
Multivariable-adjusted *β* coeff.†	Ref.	−0·004	−0·086	−0·077	0·20
Systolic blood pressure					
Participants (*n*)	88	104	98	101	
Mean (mmHg)	145·2	143·5	145·4	144·9	
sd	23·7	21·3	22·1	22·2	
Unadjusted coeff.	Ref.	−1·720	0·216	−0·271	0·89
Multivariable-adjusted *β* coeff.*	Ref.	−0·371	1·907	1·368	0·53
Multivariable-adjusted *β* coeff.†	Ref.	0·142	2·416	2·191	0·39
Diastolic blood pressure					
Participants (*n*)	88	104	98	101	
Mean (mmHg)	82·2	80·6	83·0	81·1	
sd	12·2	10·6	11·6	11·9	
Unadjusted coeff.	Ref.	−1·613	0·809	−1·032	0·92
Multivariable-adjusted *β* coeff.*	Ref.	−1·281	0·899	−0·262	0·77
Multivariable-adjusted *β* coeff.†	Ref.	0·877	1·300	0·385	0·52
Pulse pressure					
Participants (*n*)	88	104	98	101	
Mean (mmHg)	63·0	62·9	62·4	63·8	
sd	18·2	17·5	16·3	17·1	
Unadjusted ·coeff.	Ref.	−0·108	−0·593	0·761	0·81
Multivariable-adjusted *β* coeff.*	Ref.	0·910	1·008	1·630	0·53
Multivariable-adjusted *β* coeff.†	Ref.	1·019	1·117	1·805	0·50

Coeff., coefficient; ref., reference category.

* Multivariable-adjusted model 1, adjusted for age, BMI, social class (manual and non-manual workers), alcohol intake (non-drinker, drinker has been divided into three equal groups), smokers (non-smoker, current smoker, previous smoker), leisure activity (yes and no) and food energy intake.

† Multivariable-adjusted model 2, additionally adjusted for Ca intake.

‡ Original data were transformed to natural logarithms for regression model.

After over 20 years of follow-up (mean follow-up is 22·8 years), a tendency for a lower pulse pressure was seen in those with the highest vitamin D intake, but this did not reach significance (Table 5). In the analyses of the associations of SBP and DBP with vitamin D intake, DBP showed a positive correlation with vitamin D intake in the multivariate-adjusted models (adjusted model 1: *P*=0·04; adjusted model 2: *P*=0·03), but no significant associations were found between vitamin D intake and SBP.

**Table 5:** Longitudinal analysis between baseline vitamin D intake and markers of CVD risk after over 20 years of follow-up, Caerphilly Prospective Cohort Study

	Vitamin D intake from foods (µg/week)				
Characteristic	0·1–9·9	10·0–15·1	15·2–27·2	≥27·3	*P* for trend
Mean arterial pressure					
Participants (*n*)	43	43	47	41	
Mean (mmHg)	96·35	96·07	99·63	95·64	
sd	10·19	14·07	13·09	13·70	
Unadjusted coeff.	Ref.	−0·284	3·279	−0·702	0·82
Multivariable-adjusted *β* coeff.*	Ref.	0·326	3·919	1·000	0·42
Multivariable-adjusted *β* coeff.†	Ref.	0·985	4·556	1·937	0·28
Pulse wave velocity					
Participants (*n*)	43	45	47	39	
Mean (m/s)	11·89	11·40	11·77	11·48	
sd	2·61	2·82	2·71	2·78	
Unadjusted coeff.	Ref.	−0·495	−0·123	−0·411	0·66
Multivariable-adjusted *β* coeff.*	Ref.	−0·413	0·052	−0·098	0·89
Multivariable-adjusted *β* coeff.†	Ref.	−0·339	0·124	0·009	0·76
Augmentation index					
Participants (*n*)	43	46	47	41	
Mean (%)	27·35	25·30	27·04	24·70	
sd	8·49	10·68	8·81	9·26	
Unadjusted coeff.	Ref.	−2·044	−0·306	−2·654	0·36
Multivariable-adjusted *β* coeff.*	Ref.	−2·147	−0·37	−4·051	0·10
Multivariable-adjusted *β* coeff.†	Ref.	−1·389	0·363	−2·971	0·27
Systolic blood pressure					
Participants (*n*)	43	46	47	41	
Mean (mmHg)	143·3	141·9	143·7	139·8	
sd	16·2	18·6	20·9	20·3	
Unadjusted coeff.	Ref.	−1·368	0·379	−3·522	0·52
Multivariable-adjusted *β* coeff.*	Ref.	−0·055	1·225	−1·464	0·86
Multivariable-adjusted *β* coeff.†	Ref.	0·971	2·218	−0·003	0·89
Diastolic blood pressure					
Participants (*n*)	43	46	47	41	
Mean (mmHg)	72·3	72·2	76·1	74·3	
sd	9·2	11·5	10·4	12·0	
Unadjusted coeff.	Ref.	−0·128	3·847	1·966	0·17
Multivariable-adjusted *β* coeff.*	Ref.	0·141	4·378	3·574	0·04
Multivariable-adjusted *β* coeff.†	Ref.	0·456	4·683	4·023	0·03
Pulse pressure‡					
Participants (*n*)	43	46	47	41	
Mean (mmHg)	71·0	69·8	67·5	65·5	
sd	15·8	14·4	17·1	17·9	
Unadjusted coeff.	Ref.	−0·013	−0·055	−0·087	0·06
Multivariable-adjusted *β* coeff.*	Ref.	0·004	−0·047	−0·08	0·08
Multivariable-adjusted *β* coeff.†	Ref.	0·014	−0·037	−0·066	0·13

Coeff., coefficient; ref., reference category.

* Multivariable-adjusted model 1, adjusted for age, BMI, social class (manual and non-manual workers), alcohol intake (non-drinker, drinker has been divided into three equal groups), smokers (non-smoker, current smoker, previous smoker), leisure activity (yes and no) and food energy intake.

† Multivariable-adjusted model 2, additionally adjusted for Ca intake. ‡Original data were transformed to natural logarithms for regression model.

There were no significant associations between vitamin D intake and other CVD risk markers: fasting glucose/insulin (see online supplementary material, Supplemental Tables 1–3), mean arterial pressure, pulse wave velocity and augmentation index (Table 5). Also, there were no significant associations of vitamin D intake and cardiovascular events (stroke, MI, heart failure) or all-cause mortality after over 20 years of follow-up (Table 6).

**Table 6:** Longitudinal analysis between baseline vitamin D intake and CVD risk (stroke, myocardial infarction (MI), heart failure (HF) and all-cause mortality) after over 20 years of follow-up, Caerphilly Prospective Cohort Study

			Non-adjusted		Adjusted model 1*		Adjusted model 2†	
Vitamin D intake (µg/week)	No. of men	No. of strokes	OR	95 % CI	OR	95 % CI	OR	95 % CI
0·1–9·9	114	14	1·00	Ref.	1·00	Ref.	1·00	Ref.
10·0–15·1	112	21	1·65	0·79, 3·43	1·77	0·84, 3·76	1·67	0·79, 3·57
15·2–27·2	114	19	1·43	0·68, 3·01	1·58	0·74, 3·40	1·48	0·68, 3·20
≥27·3	112	18	1·37	0·64, 2·90	1·54	0·71, 3·37	1·41	0·64, 3·13
*P* for trend			0·54		0·36		0·51	
			Non-adjusted		Adjusted model 1*		Adjusted model 2†	
Vitamin D intake (µg/week)	No. of men	No. of MI	OR	95 % CI	OR	95 % CI	OR	95 % CI
0·1–9·9	114	36	1·00	Ref.	1·00	Ref.	1·00	Ref.
10·0–15·1	112	37	1·07	0·61, 1·87	1·13	0·63, 2·02	1·11	0·62, 2·00
15·2–27·2	114	35	0·96	0·55, 1·68	1·09	0·61, 1·95	1·07	0·59, 1·93
≥27·3	112	34	0·94	0·54, 1·66	1·14	0·63, 2·07	1·12	0·61, 2·05
*P* for trend			0·76		0·71		0·76	
			Non-adjusted		Adjusted model 1*		Adjusted model 2†	
Vitamin D intake (*µ*g/week)	No. of men	No. of HF	OR	95 % CI	OR	95 % CI	OR	95 % CI
0·1–9·9	114	10	1·00	Ref.	1·00	Ref.	1·00	Ref.
10·0–15·1	112	13	1·37	0·57, 3·26	1·87	0·74, 4·72	1·66	0·65, 4·24
15·2–27·2	114	10	1·00	0·40, 2·50	1·19	0·45, 3·14	1·02	0·38, 2·73
≥27·3	112	10	1·02	0·41, 2·55	1·18	0·44, 3·15	0·95	0·35, 2·59
*P* for trend			0·85		0·99		0·66	
			Non-adjusted		Adjusted model 1*		Adjusted model 2†	
Vitamin D intake (µg/week)	No. of men	No. of deaths	OR	95 % CI	OR	95 % CI	OR	95 % CI
0·1–9·9	114	76	1·00	Ref.	1·00	Ref.	1·00	Ref.
10·0–15·1	112	69	0·80	0·47, 1·38	0·84	0·46, 1·52	0·89	0·49, 1·64
15·2–27·2	114	65	0·66	0·39, 1·14	0·77	0·43, 1·39	0·82	0·46, 1·49
≥27·3	112	71	0·87	0·50, 1·50	0·98	0·53, 1·81	1·09	0·59, 2·03
*P* for trend			0·47		0·89		0·86	

Ref. reference category. OR derived from logistic regression.

* Model 1: multivariable-adjusted model adjusted for age, BMI, social class (manual and non-manual workers), alcohol intake (non-drinker, drinker has been divided into three equal groups), smokers (non-smoker, current smoker, previous smoker), leisure activity (yes and no) and food energy intake.

† Model 2: additionally adjusted for Ca intake.

## Discussion

In the present UK prospective cohort study of middle-aged men with over 20 years of follow-up, we found higher vitamin D intake was associated with lower plasma TAG concentration after cross-sectional analysis at baseline and longitudinally at the 5-year follow-up, but not at the 10-year follow-up. After over 20 years of follow-up, a modest positive association was found between vitamin D intake and DBP. In contrast, no associations were found between vitamin D and other metabolite markers or disease outcomes of stroke, MI, heart failure and all-cause mortality.

To our knowledge, the present study is the first prospective study to investigate the associations between vitamin D intake and blood lipid profile in a generally healthy population using both cross-sectional and longitudinal analyses. The negative association between vitamin D intake and plasma TAG at baseline and the 5-year examination is similar to that seen in a study^(^ ^23^ ^)^ which involved weight reduction and daily supplementation with vitamin D (83 µg) in healthy overweight subjects for over 12 months. This resulted in TAG concentration decreasing by 13·5 % in the treatment group, yet increasing by 3 % in the placebo group (*P*<0·001), and the vitamin D supplement did not adversely affect the loss of weight^(^ ^23^ ^)^. Our results are also in line with the findings of a 6-month randomised controlled trial^(^ ^24^ ^)^ in postmenopausal women with type 2 diabetes, which showed that a daily 100 µg dose of cholecalciferol (vitamin D_3_) significantly decreased the concentration of serum TAG by 1·9 mmol/l (*P*=0·02).

Several potential mechanisms for the observed inverse association of vitamin D intake with serum TAG have been proposed, namely the ability of vitamin D to modulate the growth of vascular smooth muscle cells^(^ ^25^ ^)^, antithrombotic homeostasis^(^ ^26^ ^)^ and inflammation markers of CVD^(^ ^27^ ^)^. Furthermore, previous studies^(^ ^28^ ^,^ ^29^ ^)^ have reported that vitamin D status (plasma 25-hydroxyvitamin D) is inversely associated with TAG concentration; thus, to better clarify how vitamin D status is related to CVD risk, future biomarker studies of CVD and plasma 25-hydroxyvitamin D are warranted.

However, it is not clear why the negative association between vitamin D intake and plasma TAG was found at 5 years, but not after 10 years in the longitudinal analysis. One possible explanation may be dietary change during the follow-up period. One study^(^ ^30^ ^)^ showed that there has been a trend towards lower fat content in the UK diet since the 1980s. As vitamin D is a fat-soluble vitamin^(^ ^1^ ^)^, it is likely that vitamin D intake has also declined and indeed the current study showed vitamin D intake to be positively associated with fat intake. Therefore the lack of association between vitamin D intake and TAG concentration after 10 years may be due to vitamin D intake declining over that period. In addition, we believe our study is the first to show a significant positive cross-sectional association between vitamin D intake and pulse pressure, although no association was seen in the longitudinal analysis. A positive association between vitamin D intake and DBP after over 20 years of follow-up was observed, although no association was found for SBP. This needs confirmation in further studies.

There are very few studies that have reported associations between vitamin D intake and CVD risk or all-cause mortality. Our null finding of vitamin D intake was consistent with earlier prospective studies^(^ ^12^ ^,^ ^13^ ^)^. The Iowa Women’s Health Study (WHS) in 1999^(^ ^12^ ^)^ similarly found no association between vitamin D intake and IHD mortality over an 8-year follow-up period and the study of Bonthuis *et al*.^(^ ^13^ ^)^ also reported vitamin D intake not to be associated with CVD mortality or all-cause mortality during 14·4 years of follow-up. Furthermore, our study agrees with a systematic review of fifty-six randomised controlled trails, which did not find a significant association between vitamin D supplementation and total mortality risk^(^ ^31^ ^)^. However, vitamin D intake (4·30 (sd 3·3) µg/d) was reported in another 10-year follow-up prospective study of 361 men and 394 women^(^ ^10^ ^)^ and suggested a protective role of vitamin D intake on stroke, but not MI. The contrasting findings of these studies may be due to the different characteristics of the study participants. For example, the initial mean ages of the subjects in the CAPS (mean age of 51·7 years) and Iowa WHS^(^ ^12^ ^)^ (mean age of 53·8 years) were similar, but age was higher (range 65–99 years) in the investigation of Marniemi *et al*.^(^ ^10^ ^)^. Moreover, the study of Sun *et al*.^(^ ^11^ ^)^ showed vitamin D intake to be associated with a lower risk of CVD in men, which may be because the subjects in that study^(^ ^11^ ^)^ had higher mean vitamin D intake of 6 µg/d than subjects in the current study (3 µg/d). Therefore, further research is needed to confirm the protective effect or otherwise of higher vitamin D intake on CVD risk. In addition, whether there is a sex-specific association between vitamin D intake and CVD risk needs further investigation.

The recent report of the UK Scientific Advisory Committee on Nutrition^(^ ^1^ ^)^ recommended a daily Reference Nutrient Intake (RNI) of 10 µg vitamin D for the general population aged 4 years and above, including pregnant and lactating women. In CAPS, only eleven out of 452 subjects achieved the current RNI dose. The mean vitamin D intake of 3 µg/d in the current study agrees well with the most recent published data from the UK National Diet and Nutrition Survey^(^ ^32^ ^)^, which reported the mean daily vitamin D intake of 3·1 µg for men aged 19–64 years. Thus, vitamin D-fortified foods need to be considered as a possible strategy to facilitate population intake of the RNI. Furthermore, a few recent studies have used higher doses of vitamin D in their intervention trials^(^ ^33^ ^–^ ^35^ ^)^, which also showed no associations of vitamin D intake with CVD risk.

The strength of the CAPS is the long (over 20 years) follow-up period. The current novel study presents both cross-sectional and longitudinal relationships between vitamin D intake and CVD risk. The longitudinal analysis was conducted at 5 years, 10 years and over 20 years, which provides the opportunity to test the consistency of the influence of vitamin D intake on CVD events. There are, however, several limitations of the study. One potential limitation is that vitamin D intake was assessed at baseline only and was not repeated during the follow-up period to assess the extent of any diet change. Moreover, CVD risk markers were not measured at the 10-year follow-up. A further limitation in the present study was that the results apply to men only, which may not represent associations in women. Finally, unknown residual confounding factors may have influenced the outcomes seen. In particular, vitamin D status of the subjects was not measured initially or during the follow-up of CAPS and there were no assessments of sunshine exposure; thus, it was not possible to investigate the correlation between vitamin D status and vitamin D dietary intake. In addition, because the relatively small cohort size of the current study may not be representative all UK men, further prospective studies with large subject numbers would provide more evidence on effect of the vitamin D intake on CVD risk and/or all-cause mortality and possible mechanisms of action.

## Conclusions

The current investigation from CAPS, a prospective cohort study, provides further evidence about the potential benefit of vitamin D intake on circulating TAG concentration. Additional studies are needed to verify the current finding, especially randomised controlled intervention trials on the effect of vitamin D intake on CVD risk markers in subjects of low vitamin D status and mechanistic studies to determine the mode of action.

## Supplementary material

For supplementary material accompanying this paper visit https://doi.org/10.1017/S1368980017001732. click here to view supplementary material
