# Supplementary material for: Vitamin D intake and risk of CVD and all-cause mortality: evidence from the Caerphilly Prospective Cohort Study
Source: Public Health Nutr. 2017 Aug 14;20(15):2744–53. doi: 10.1017/S1368980017001732 (PMC10261356; doi:10.1017/S1368980017001732)
Supplement: Supplementary file 1 [file S1368980017001732sup001.docx]

| **Supplemental Table 1.** Cross-sectional analysis between baseline vitamin D intake and plasma glucose and insulin | | | | | | | | | |
| --- | --- | --- | --- | --- | --- | --- | --- | --- | --- |
|  | Vitamin D intake from foods | | | | | | | |  |
| Characteristics | 0.1-9.9 µg/wk | | 10-15.1 µg/wk | | 15.2-27.2 µg/wk | | ≥27.3 µg/wk | | *P* for trend |
| Fasting glucose |  |  |  |  |  |  |  |  |  |
| Participants, n | 113 |  | 113 |  | 113 |  | 113 |  |  |
| Mean (SD) , mmol/L | 4.90 | (1.02) | 5.07 | (1.81) | 4.86 | (0.66) | 4.93 | (1.17) |  |
| Unadjusted Coef. | ref |  | 0.171 |  | -0.038 |  | 0.024 |  | 0.79 |
| Multivariable-adjusted β-coef^*^ | ref |  | 0.174 |  | -0.022 |  | 0.068 |  | 0.99 |
| Multivariable-adjusted β-coef† | ref |  | 0.176 |  | -0.019 |  | 0.071 |  | 0.99 |
| Insulin‡ |  |  |  |  |  |  |  |  |  |
| Participants, n | 92 |  | 104 |  | 101 |  | 104 |  |  |
| Mean (SD), mmol/L | 1.08 | (2.24) | 1.09 | (3.1) | 1.07 | (4.32) | 0.76 | (0.88) |  |
| Unadjusted Coef. | ref |  | 0.037 |  | -0.128 |  | -0.086 |  | 0.23 |
| Multivariable-adjusted β-coef^*^ | ref |  | 0.043 |  | -0.124 |  | -0.042 |  | 0.41 |
| Multivariable-adjusted β-coef† | ref |  | 0.056 |  | -0.109 |  | -0.02 |  | 0.52 |

^*^Multivariable-adjusted model adjusted for age, BMI, social class (manual and non-manual workers), alcohol intake (non-drinker, drinker has been divided into 3 equal groups), smokers (non-smoker, current smoker, previous smoker), leisure activity (yes and no), food energy intake.

†Additionally adjusted for calcium intake.

‡original data were transformed to natural logarithms for regression model.

| **Supplemental Table 2.** Longitudinal analysis between baseline vitamin D intake and plasma glucose and insulin after 5-years of follow-up. | | | | | | | | | |
| --- | --- | --- | --- | --- | --- | --- | --- | --- | --- |
|  | Vitamin D intake from foods | | | | | | | |  |
| Characteristics | 0.1-9.9 µg/wk | | 10-15.1 µg/wk | | 15.2-27.2 µg/wk | | ≥27.3 µg/wk | | *P* for trend |
| Fasting glucose |  |  |  |  |  |  |  |  |  |
| Participants, n | 109 |  | 110 |  | 111 |  | 109 |  |  |
| Mean (SD), mmol/L | 5.39 | (1.09) | 5.63 | (2.03) | 5.14 | (0.64) | 5.35 | (1.28) |  |
| Unadjusted Coef. | ref |  | 0.243 |  | -0.247 |  | -0.036 |  | 0.30 |
| Multivariable-adjusted β-coef^*^ | ref |  | 0.307 |  | -0.231 |  | -0.018 |  | 0.32 |
| Multivariable-adjusted β-coef† | ref |  | 0.322 |  | -0.216 |  | 0.004 |  | 0.36 |
| Insulin^4^ |  |  |  |  |  |  |  |  |  |
| Participants, n | 49 |  | 43 |  | 52 |  | 47 |  |  |
| Mean (SD), mmol/L | 2.85 | (2.12) | 3.7 | (1.76) | 3.42 | (2.99) | 3.54 | (2.35) |  |
| Unadjusted Coef. | ref |  | 0.501 |  | 0.313 |  | 0.361 |  | 0.05 |
| Multivariable-adjusted β-coef^*^ | ref |  | 0.481 |  | 0.28 |  | 0.358 |  | 0.06 |
| Multivariable-adjusted β-coef† | ref |  | 0.463 |  | 0.225 |  | 0.314 |  | 0.13 |

^*^Multivariable-adjusted model adjusted for age, BMI, social class (manual and non-manual workers), alcohol intake (non-drinker, drinker has been divided into 3 equal groups), smokers (non-smoker, current smoker, previous smoker), leisure activity (yes and no), food energy intake.

†Additionally adjusted for calcium intake.

‡original data were transformed to natural logarithms for regression model.

| **Supplemental Table 3.** Longitudinal analysis between baseline vitamin D intake and plasma glucose after 10-years of follow-up^1^. | | | | | | | | | |
| --- | --- | --- | --- | --- | --- | --- | --- | --- | --- |
|  | Vitamin D intake from foods | | | | | | | |  |
| Characteristics | 0.1-9.9 µg/wk | | 10-15.1 µg/wk | | 15.2-27.2 µg/wk | | ≥27.3 µg/wk | | *P* for trend |
| Fasting glucose |  |  |  |  |  |  |  |  |  |
| Participants, n | 86 |  | 96 |  | 99 |  | 99 |  |  |
| Mean (SD), mmol/L | 5.49 | (1.22) | 5.73 | (1.82) | 5.74 | (1.98) | 5.86 | (1.95) |  |
| Unadjusted Coef. | ref |  | 0.238 |  | 0.245 |  | 0.372 |  | 0.18 |
| Multivariable-adjusted β-coef^*^ | ref |  | 0.238 |  | 0.241 |  | 0.443 |  | 0.12 |
| Multivariable-adjusted β-coef† | ref |  | 0.246 |  | 0.25 |  | 0.458 |  | 0.11 |

^*^Multivariable-adjusted model adjusted for age, BMI, social class (manual and non-manual workers), alcohol intake (non-drinker, drinker has been divided into 3 equal groups), smokers (non-smoker, current smoker, previous smoker), leisure activity (yes and no), food energy intake.

^†^Additionally adjusted for calcium intake.

‡original data were transformed to natural logarithms for regression model.
